# Supplementary material for: CpG Methylation Altered the Stability and Structure of the i-Motifs Located in the CpG Islands
Source: Int J Mol Sci. 2022 Jun 9;23(12):6467. doi: 10.3390/ijms23126467 (PMC9223787; doi:10.3390/ijms23126467)
Supplement: Supplementary file 1 [file ijms-23-06467-s001.zip › ijms-1762667-supplementary.pdf]

Supplementary information

CpG methylation altered the stability and the structure of i-motifs in CpG islands

Daiki Oshikawa, Shintaro Inaba, Yudai Kitagawa, Kaori Tsukakoshi, and Kazunori Ikebukuro

Table S1. Oligonucleotides used in this study. The methylated cytosines are shown in red.

**i-motif**	Sequence (5' to 3')
VEGF	CCCCGCCCCCGGCCCGCCCC
C-KIT	GCCGGCCACGCCCCTCCTCGCC
BCL2	GCTCCCGCCCCCTTCCTCCCGCGCCCG
HRAS1	CGCCCGTGCCCTGCGCCCGCAACCCGA
HRAS2	CGCCCCCGCCCCCGCCCCGCCCCG
**G4**	Sequence (5' to 3')
HRAS2	CGGGGCGGGGCGGGGGCGGGGGCG

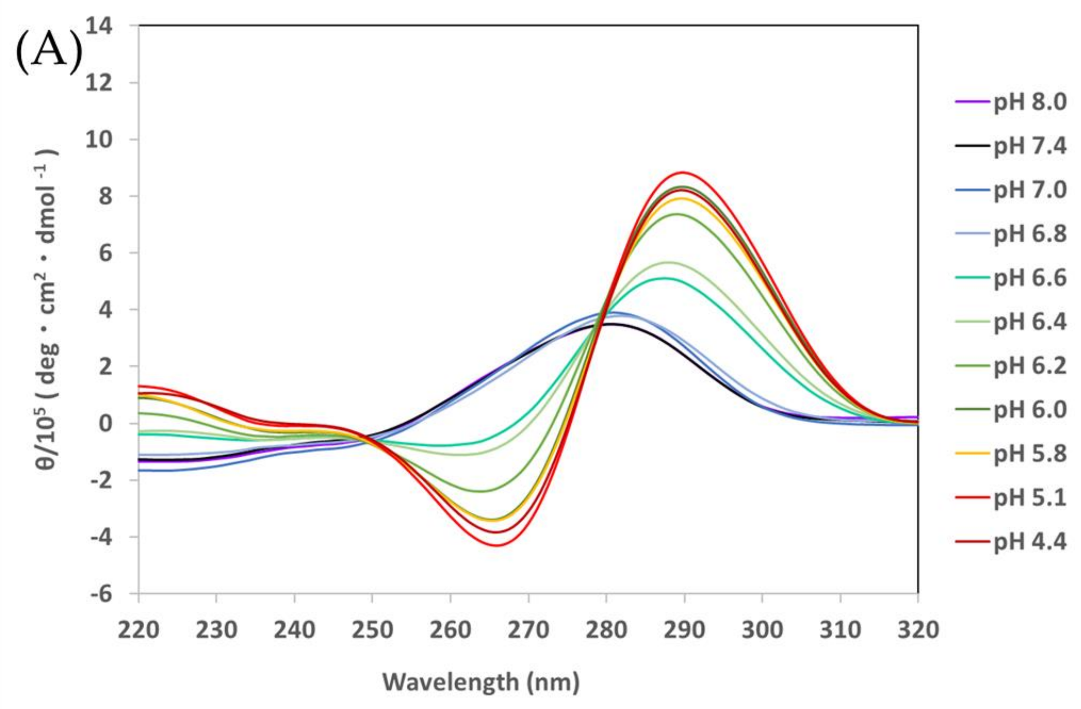

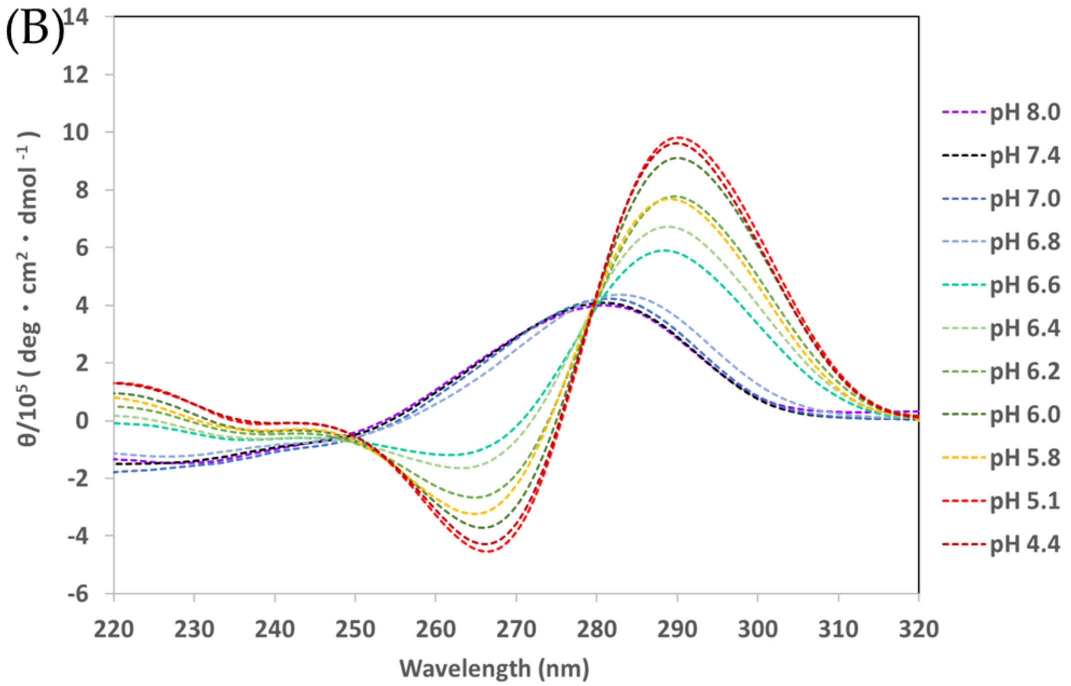

Figure S1. CD spectrum of (A) unmethylated and (B) methylated *VEGF* i-motif

(A)

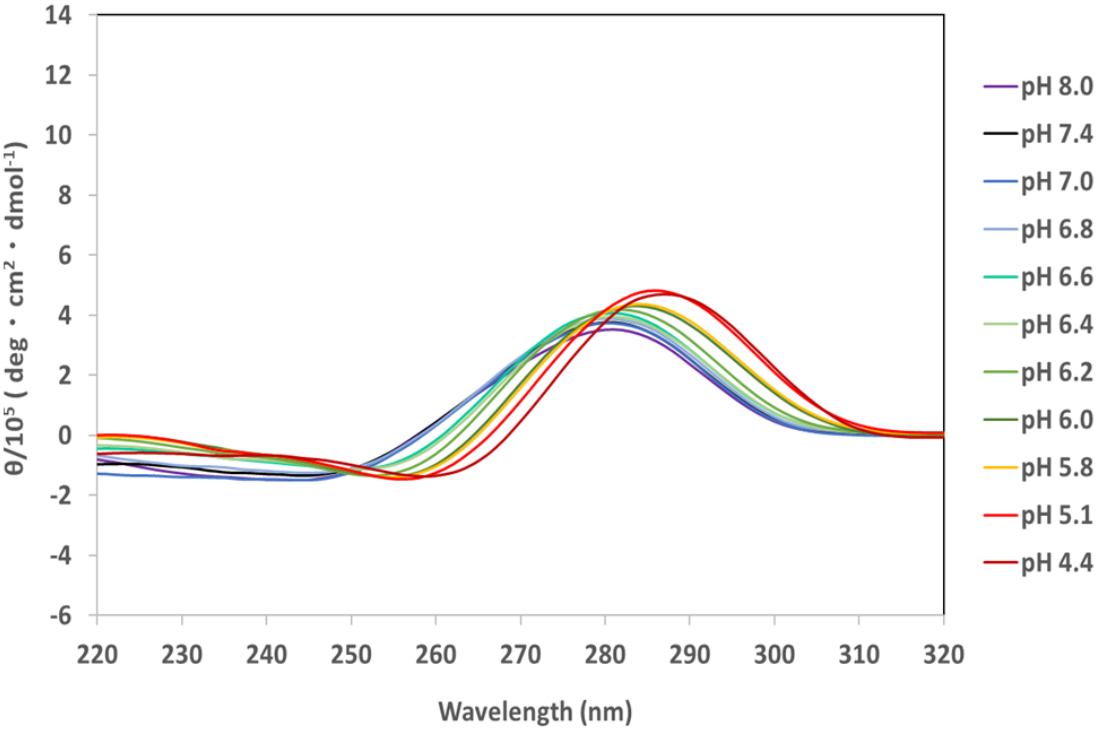

(B)

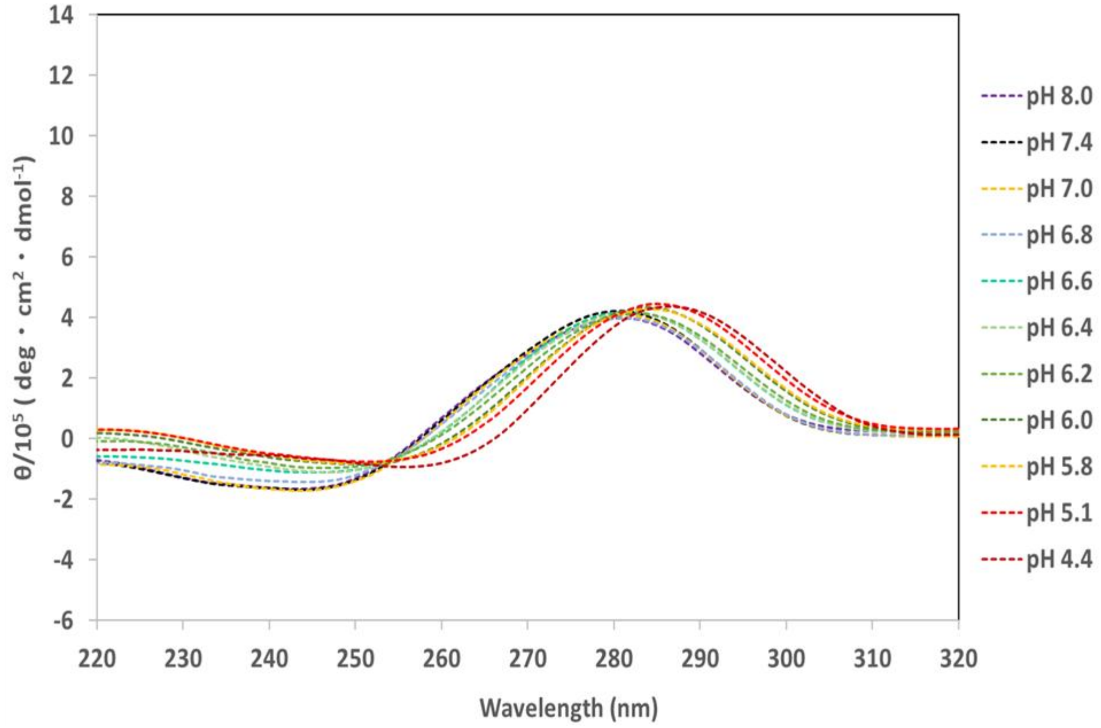

Figure S2. CD spectrum of (A) unmethylated and (B) methylated *C-KIT* i-motif

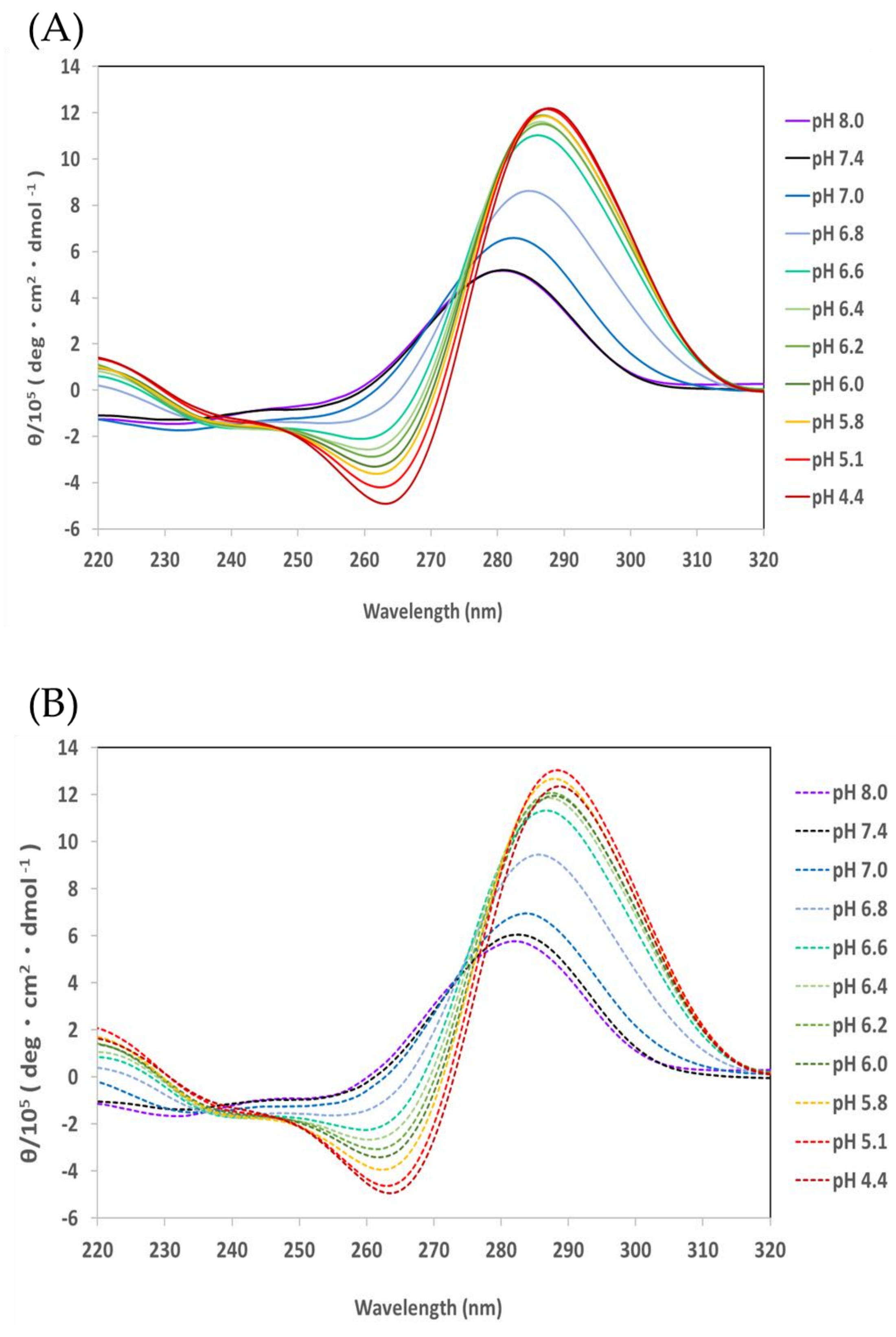

Figure S3. CD spectrum of (A) unmethylated and (B) methylated *BCL2* i-motif

(A)

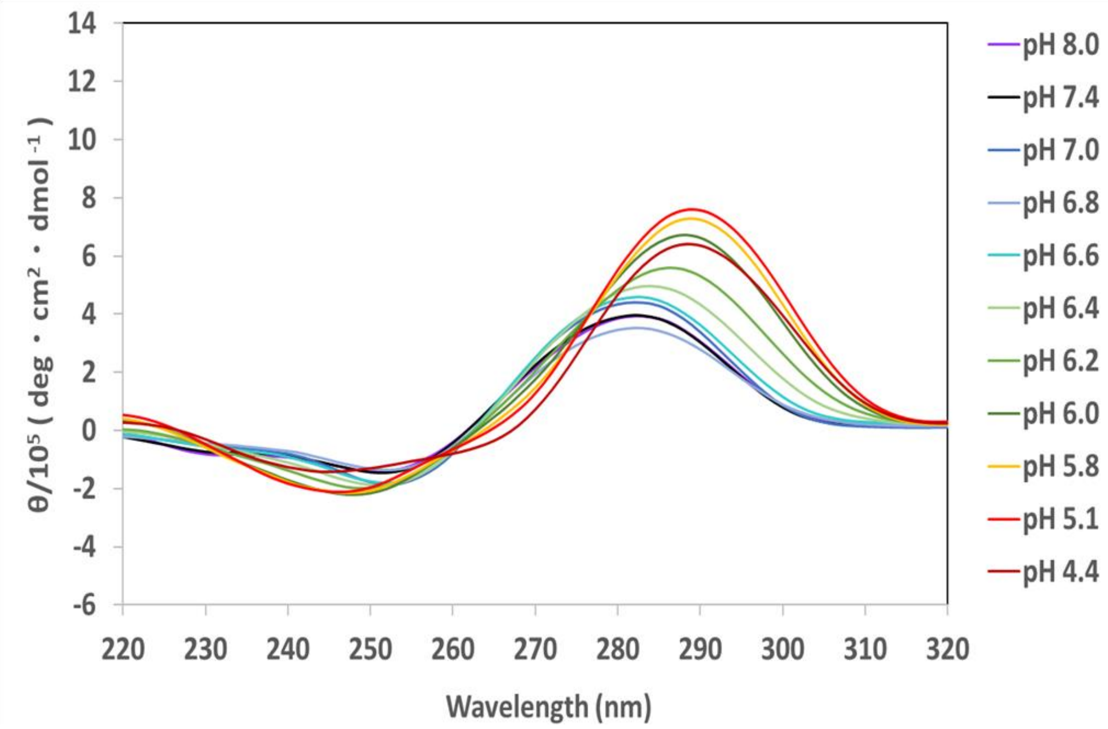

(B)

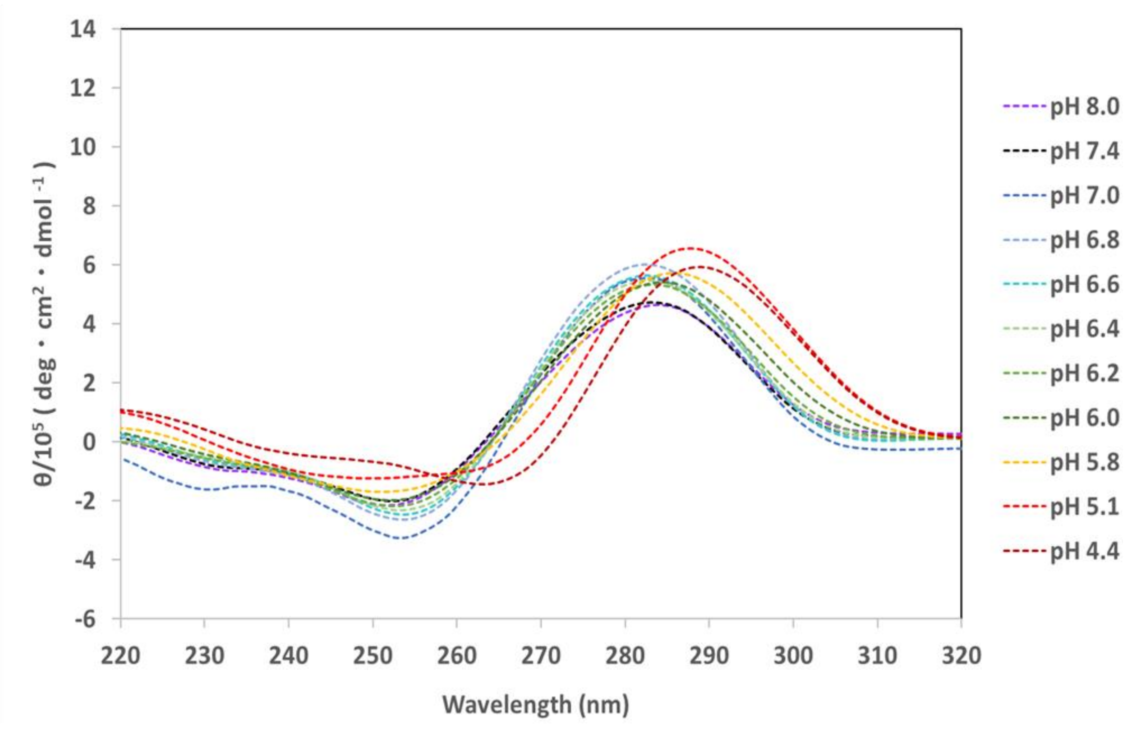

Figure S4. CD spectrum of (A) unmethylated and (B) methylated *HRAS1* i-motif

(A)

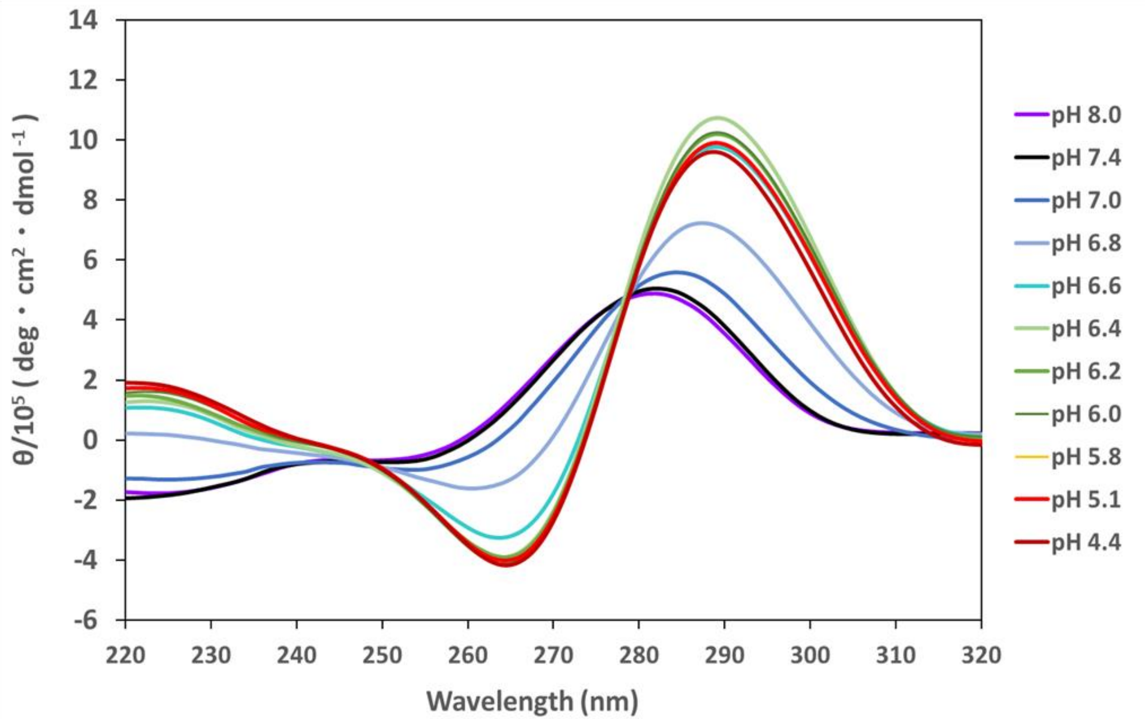

(B)

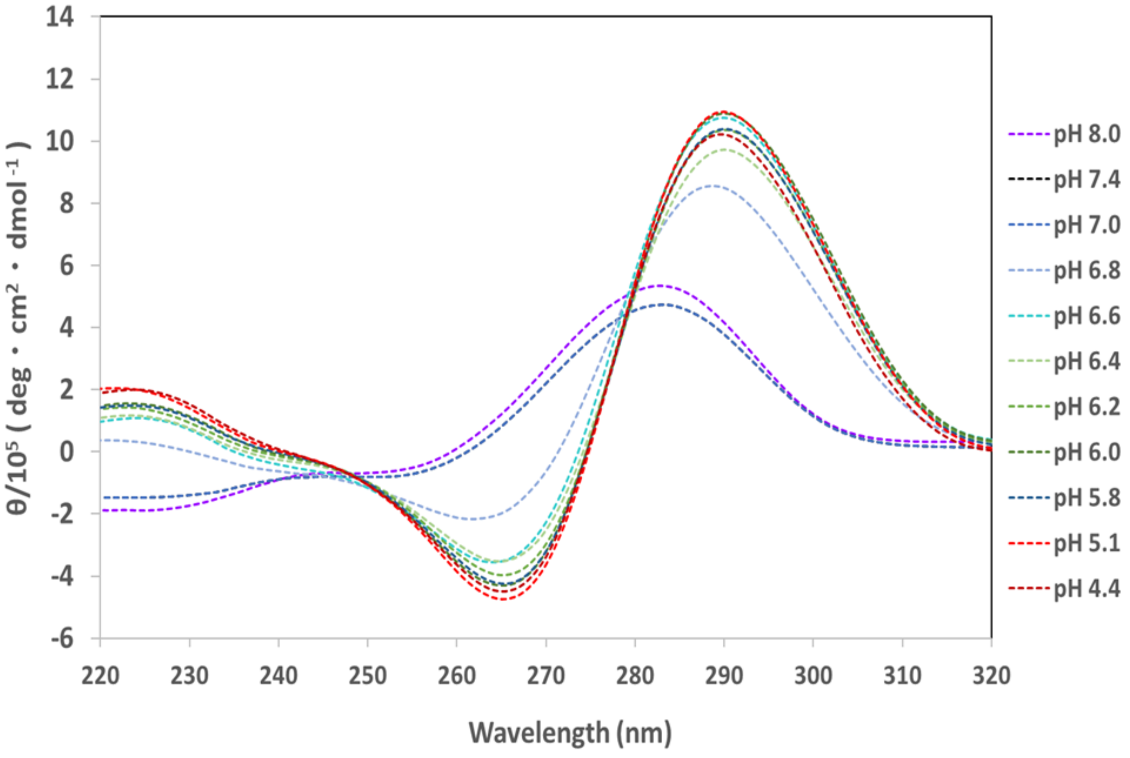

Figure S5. CD spectrum of (A) unmethylated and (B) methylated *HRAS2* i-motif

(A)

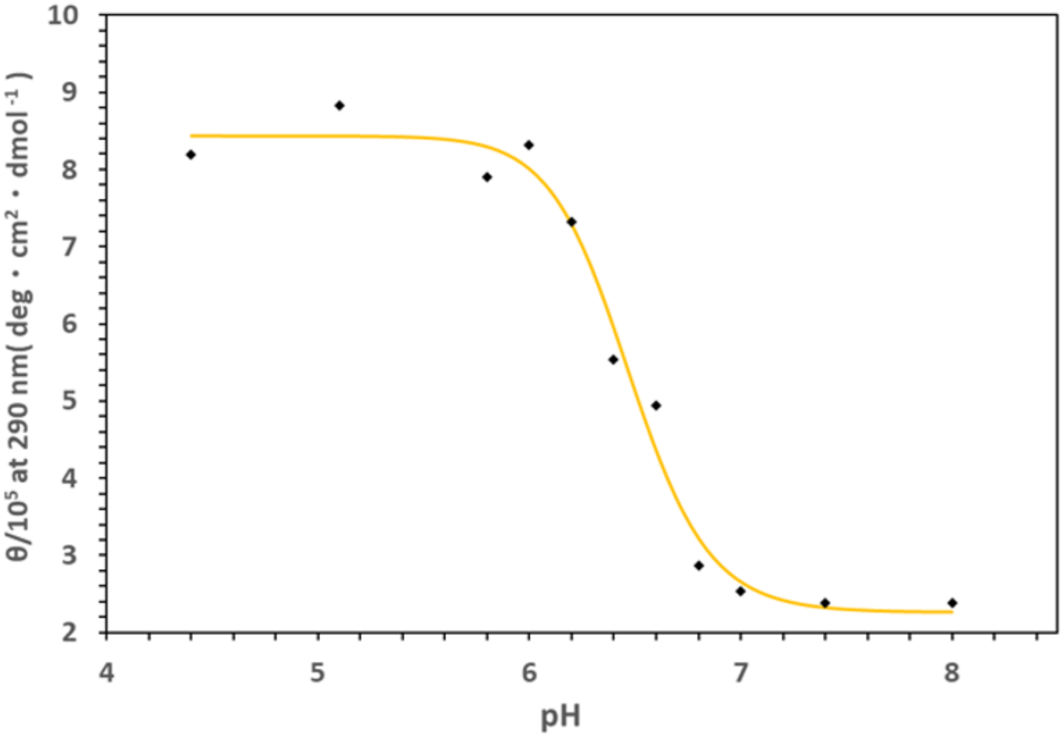

(B)

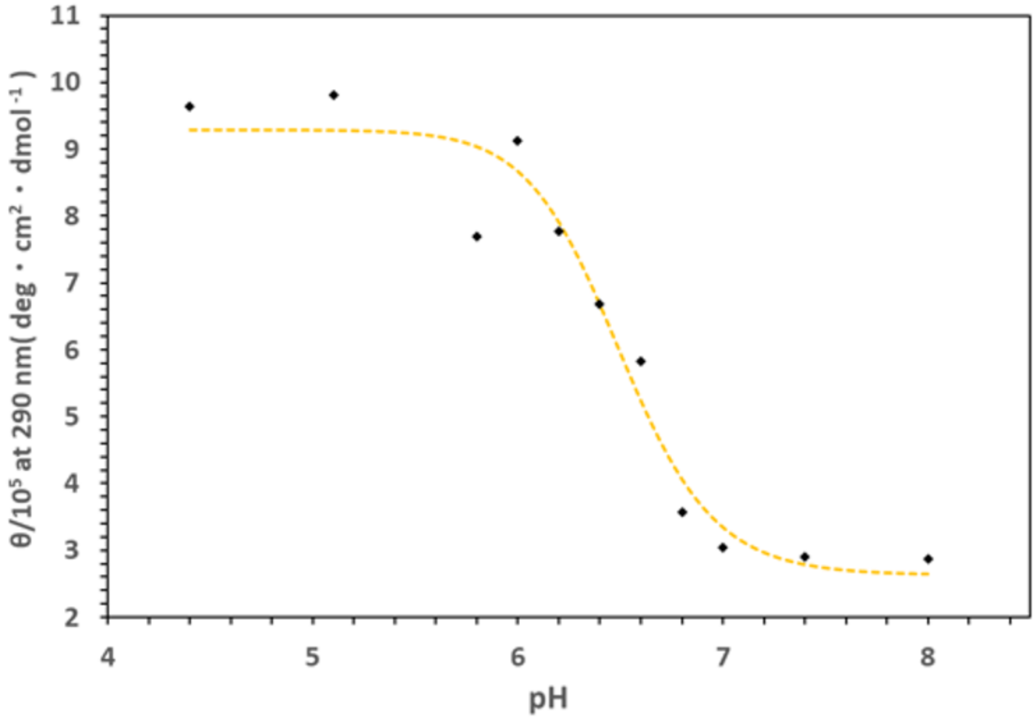

Figure S6. Molar ellipticities at 290 nm in the CD spectrum of (A) unmethylated and (B) methylated *VEGF* i-motif

(A)

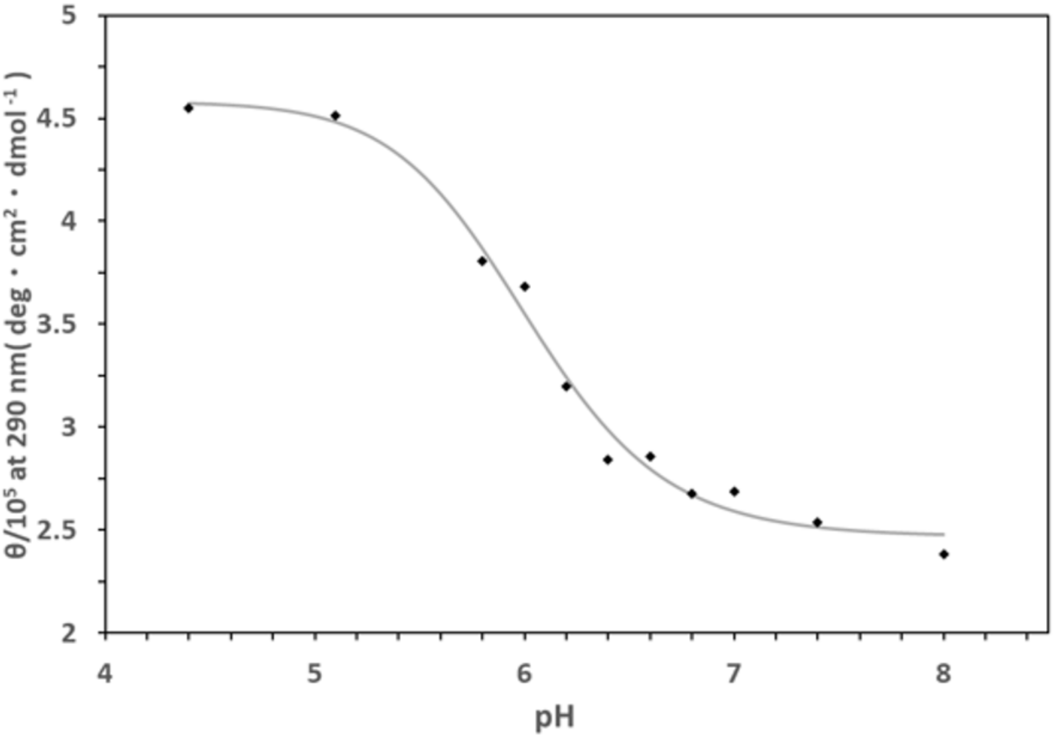

(B)

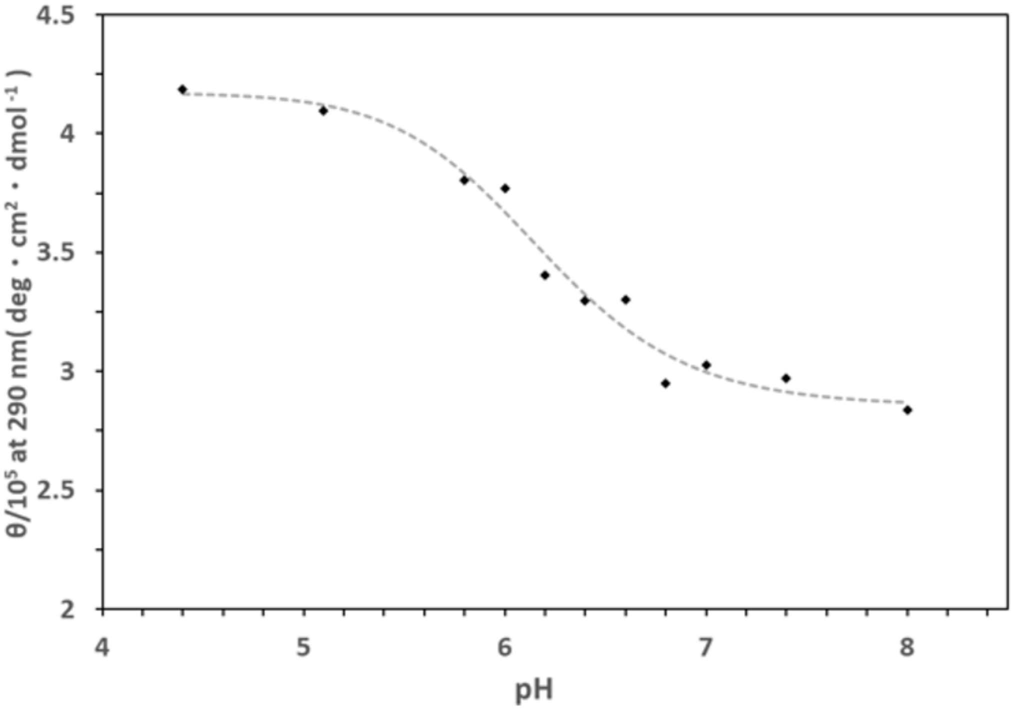

Figure S7. Molar ellipticities at 290 nm in the CD spectrum of (A) unmethylated and (B) methylated *C-KIT i*-motif

(A)

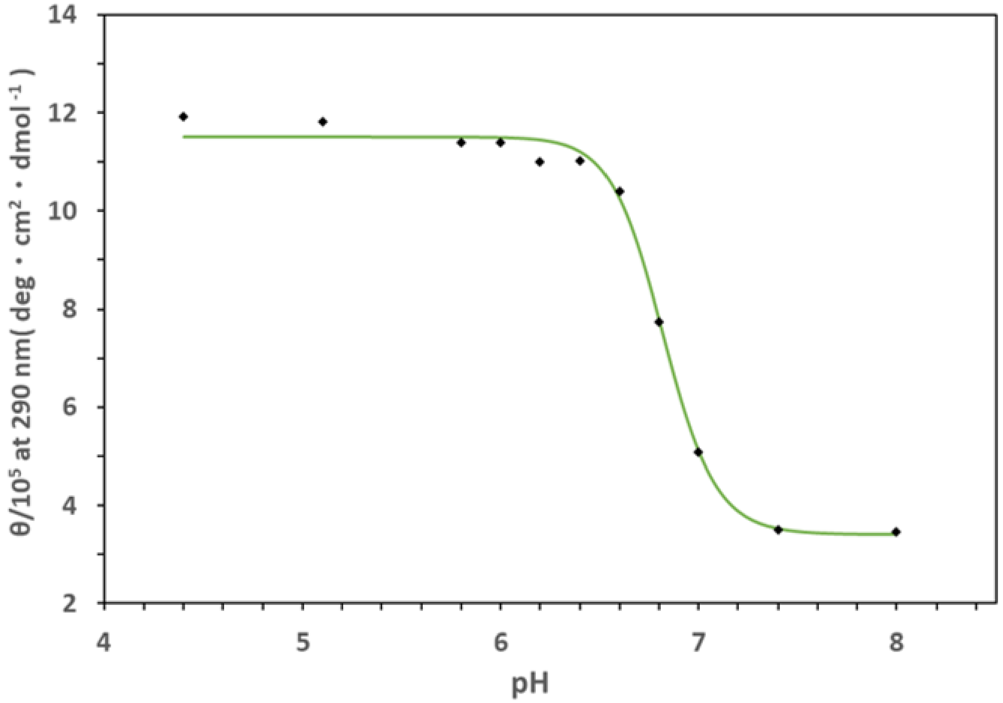

(B)

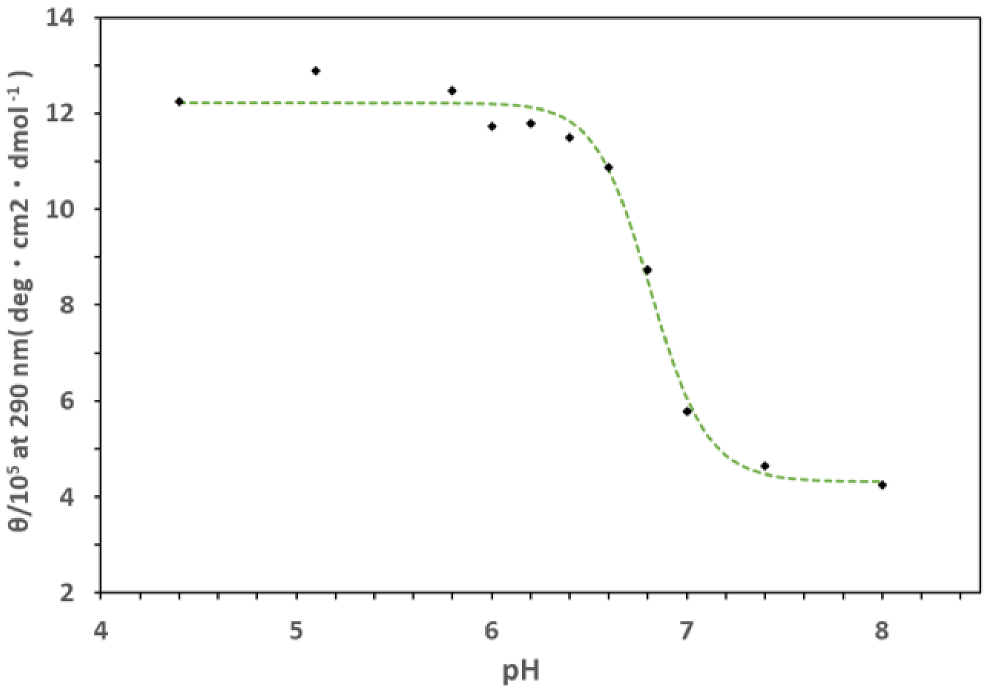

Figure S8. Molar ellipticities at 290 nm in the CD spectrum of (A) unmethylated and (B) methylated *BCL2* i-motif

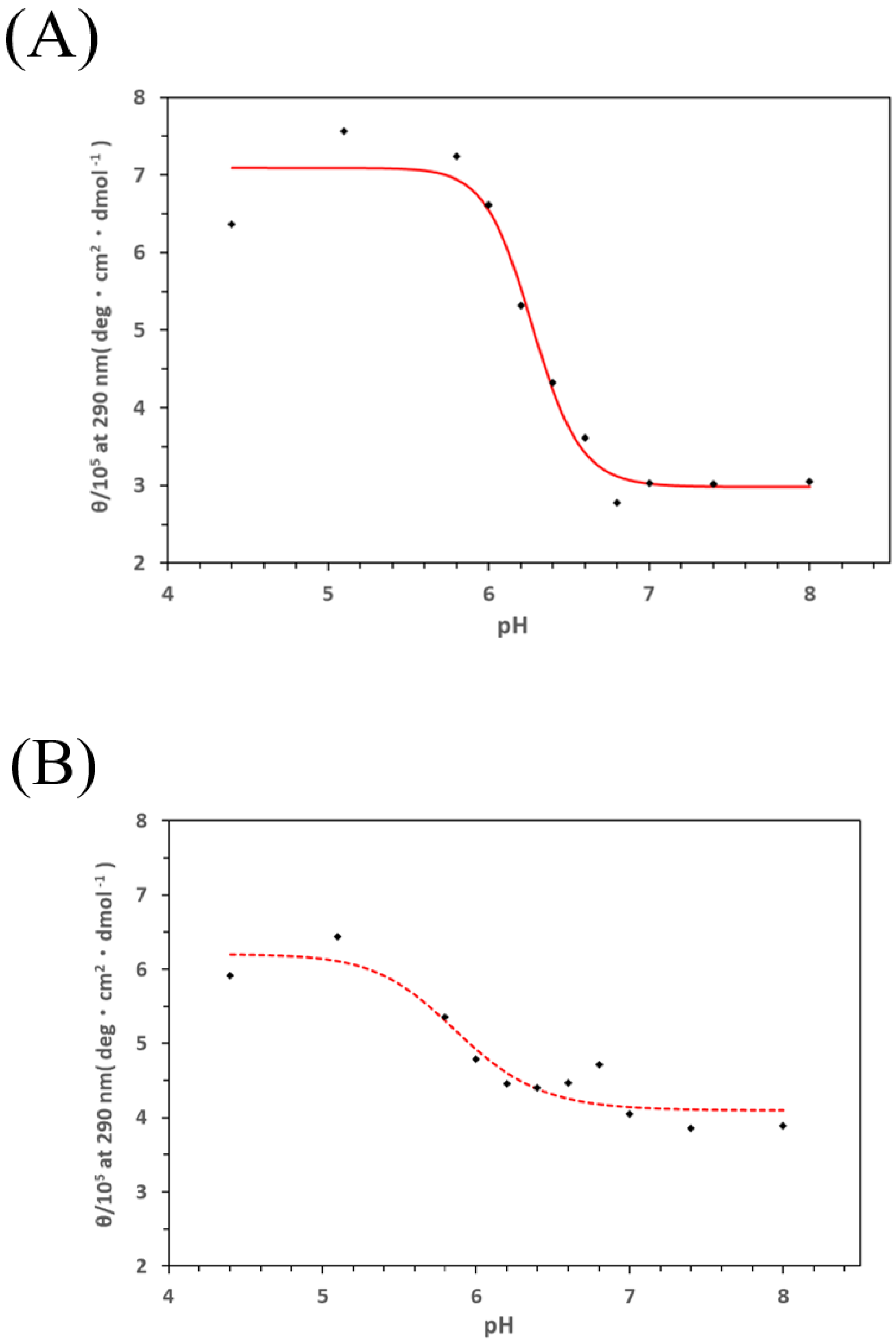

Figure S9. Molar ellipticities at 290 nm in the CD spectrum of (A) unmethylated and (B) methylated *HRAS1* i-motif

(A)

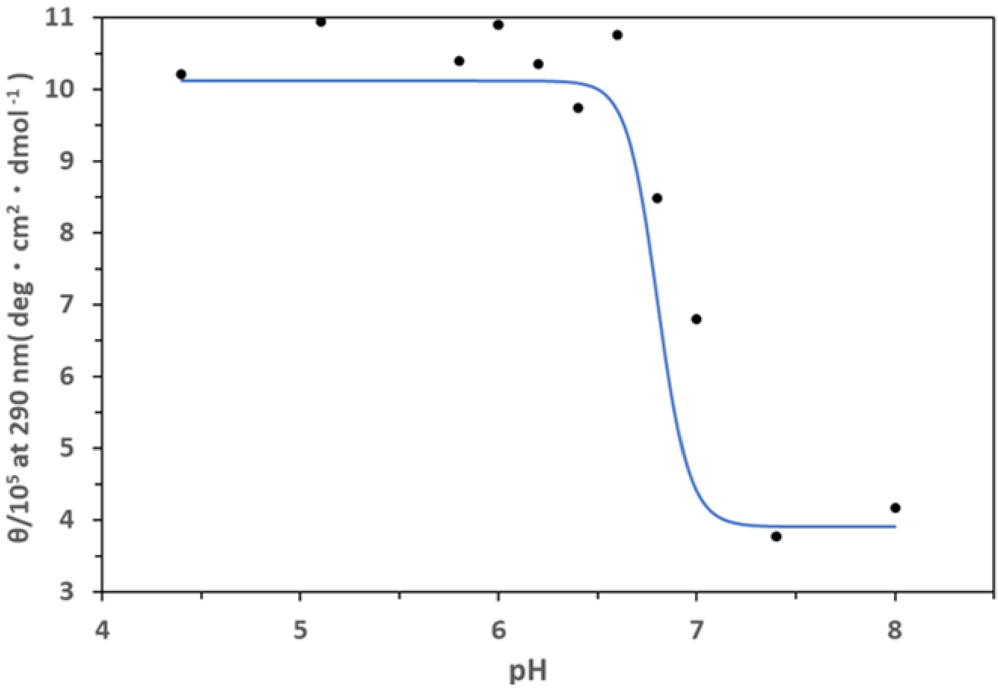

(B)

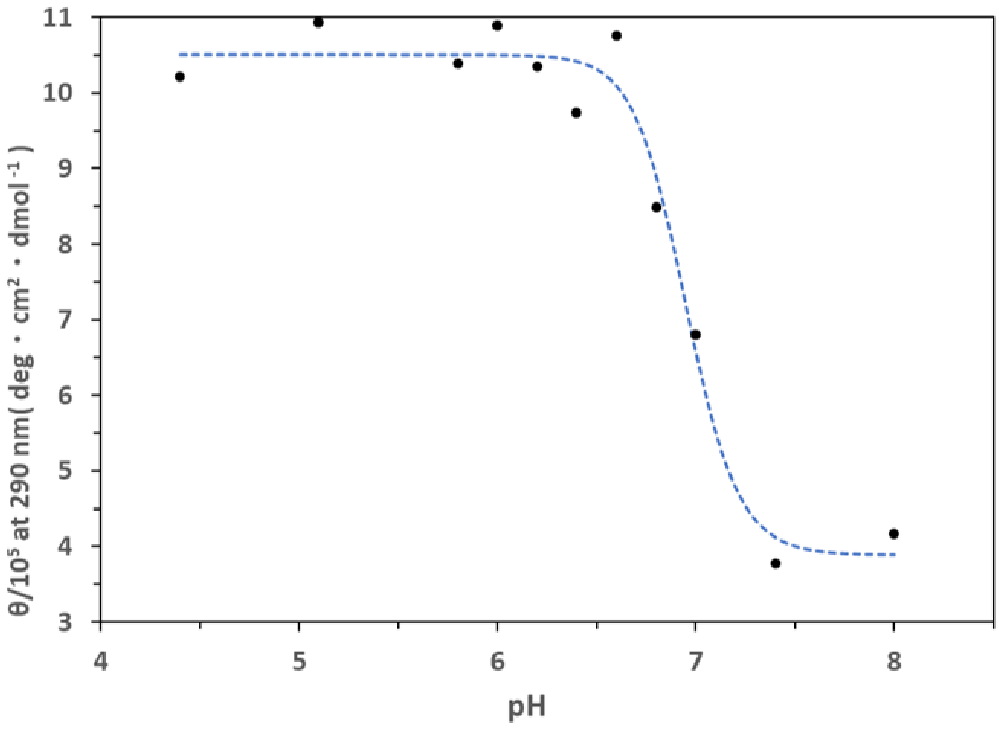

Figure S10. Molar ellipticities at 290 nm in the CD spectrum of (A) unmethylated and (B) methylated *HRAS2* i-motif

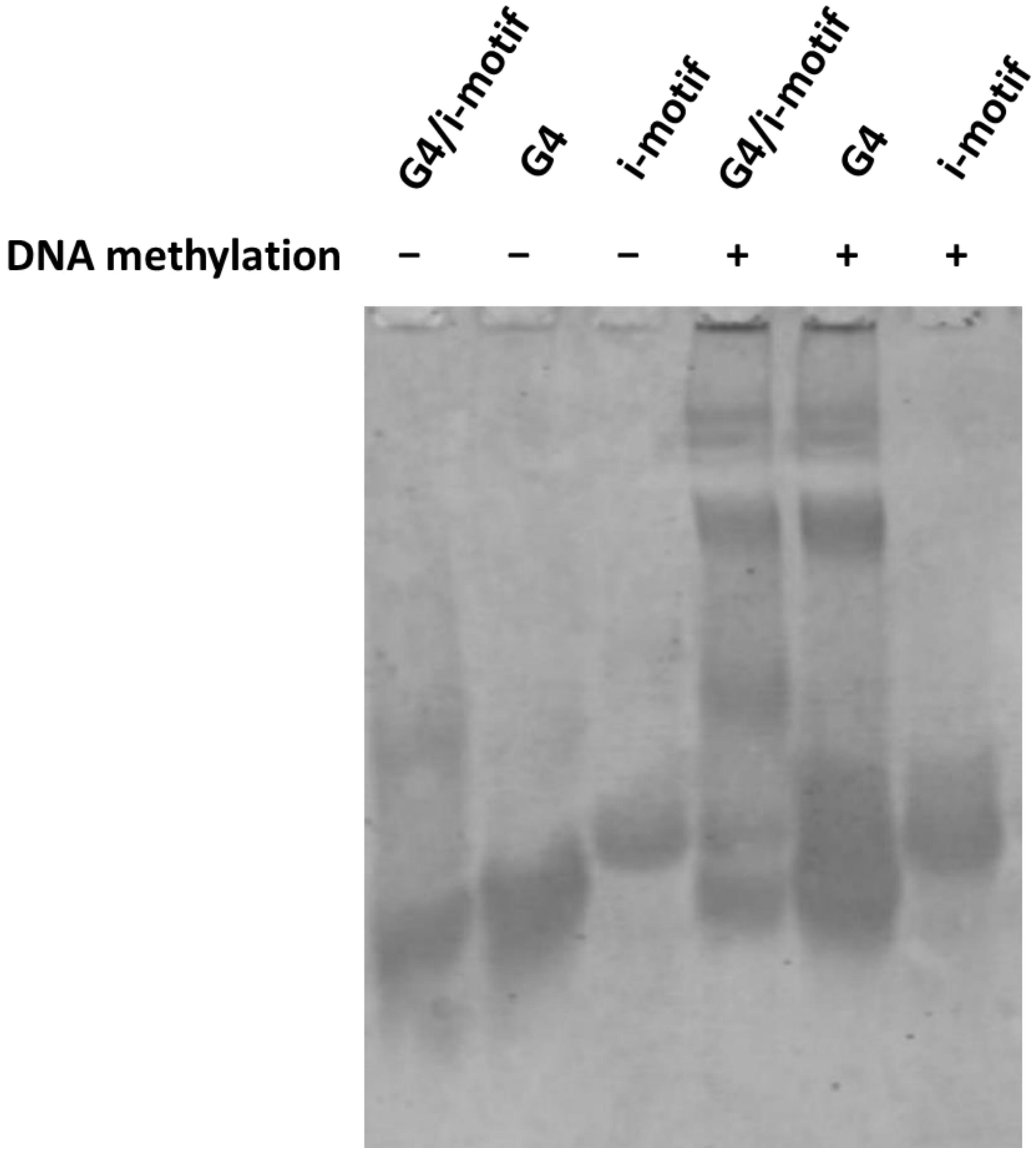

Figure S11. Native-PAGE of *HRAS2* G4/i-motif mixed DNAs, G4 DNAs, and i-motif DNAs at pH 5.8.
